# Supplementary material for: Confirming validity of The Fear of COVID-19 Scale in Japanese with a nationwide large-scale sample
Source: PLoS One. 2021 Feb 10;16(2):e0246840. doi: 10.1371/journal.pone.0246840 (PMC7875410; doi:10.1371/journal.pone.0246840)
Supplement: S1 File — (PDF) [file pone.0246840.s001.pdf]

新型コロナウイルス恐怖尺度について

この度筑波大学災害・地域精神医学では、Ahorsu DK らが開発した、新型コロナウイルス恐怖尺度（Fear of Coronaviruss-19 Scale： FCV-19S）について、原著者の承諾を得て翻訳し、日本語版を作成しました。

本尺度は、コロナに対する恐怖を定量化できることから、臨床で用いる有用性が高いと考えます。現在のコロナ禍において、本尺度の社会的意義やニーズは高いと考え、社会貢献を目的に公開いたします。自由にご利用ください。

なお、使用にあたっては以下の点にご留意ください。

（尺度の利用方法）

７つの質問項目について、それぞれ「全くあてはまらない」から「とてもあてはまる」までの５つの回答選択肢の中からあてはまる番号を選んで〇をつけてください。各質問の得点は〇をつけた番号で、最小１点、最大５点となります。総得点は７つの質問項目の〇を付けた番号の数字を合計して算出します（総得点の範囲は７点～35 点）。得点が高いほど、新型コロナウイルスの恐怖が強いことを示します。

（利用上の留意点）

作成した日本語版の妥当性や信頼性、カットオフポイントなどについてまだ検証はできていません。結果はあくまで参考にとどめてください。

（尺度の原版文献）

本尺度の原版は英語で、オープンアクセスで誰もが利用可能となっています。原尺度の掲載論文は以下です。

Daniel Kwasi Ahorsu, Chung-Ying Lin, Vida Imani, Mohsen Saffari, Mark D. Griffiths, Amir H. Pakpour: The Fear of COVID-19 Scale: Development and Initial Validation. International Journal of Mental Health and Addiction, 2020 Mar 27:1-9. doi: 10.1007/s11469-020-00270-8

（日本語版尺度作成者：太刀川弘和，根本清貴，田口高也，高橋あすみ，小川貴史，白鳥裕貴，高橋晶，doi: 10.13140/RG.2.2.32619.77605）

新型コロナウイルス（COVID-19）恐怖尺度

(Fear of Coronaviruss-19 Scale： FCV-19S 日本語版)

Ahorsu DK, Lin CY, Imani V, et al: International Journal of Mental Health and Addiction (2020)

		全くあてはまらない	あてはまらない	どちらでもない	あてはまる	とてもあてはまる
1	新型コロナウイルスがとても恐い	1	2	3	4	5
2	新型コロナウイルスについて考えると不快になる	1	2	3	4	5
3	新型コロナウイルスについて考えると手汗をかく	1	2	3	4	5
4	新型コロナウイルスで命を失うことを恐れている	1	2	3	4	5
5	インターネットで新型コロナウイルスのニュースや話題をみると、緊張したり、不安になったりする	1	2	3	4	5
6	新型コロナウイルス感染が心配で眠れない	1	2	3	4	5
7	新型コロナウイルス感染について考えると、心拍が早くなったり、動悸がしたりする	1	2	3	4	5

日本語訳：筑波大学　災害・地域精神医学（2020）
