# Supplementary material for: Confirming validity of The Fear of COVID-19 Scale in Japanese with a nationwide large-scale sample
Source: PLoS One. 2021 Feb 10;16(2):e0246840. doi: 10.1371/journal.pone.0246840 (PMC7875410; doi:10.1371/journal.pone.0246840)
Supplement: Attachment — Submitted filename: Response to Reviewers21.01.26.docx [file pone.0246840.s003.docx]

January 26, 2021

Dear Editors:

Please accept for re-submission our manuscript PONE-D-20-37753 titled “Confirming Validity of The Fear of COVID-19 Scale in Japanese with a Nationwide Large-Scale Sample.”

We thank the reviewer for the valuable suggestions which greatly improved the quality of the manuscript. Responses to all comments are below. Changes to the manuscript are highlighted.

Sincerely,

Hirokazu Tachikawa, MD, PhD

Department of Psychiatry,

Faculty of Medicine, University of Tsukuba,

1-1-1 Tennoudai, Tsukuba, Ibaraki, 305-8575, Japan.

tachikawa@md.tsukuba.ac.jp

**Answer to Review #1**

Abstract: Sampling procedure is not clear in abstract. Please add study time. Like Bitan et al I would suggest to rename the factors as: Emotional fear reactions and Symptomatic expressions of fear

🡪 Thank you very much for this suggestion. We have added the details of sampling in the abstract (p.2, ll. 23-25) and renamed the factors as *emotional fear reactions* and *symptomatic expressions of fear* (p.2, l. 27). We also added that the names of these factors were taken from the study by Bitan et al (p.15, ll. 223-224).

Introduction: I think that it will be great if the authors can provide some information regarding how Japan react to the COVID-19 (e.g., government' policies, infected cases, deaths). It would be better to justify why a scale should be translated into other languages. Please update your references on recent publication of Fear validation:

🡪 Authors greatly appreciate this suggestion to improve the Introduction. We have added a brief note on Japan's response to COVID-19, explained why FCV-19S should be translated into other languages, and updated the references on FCV-19S (p.3 ll. 50-59, p.4 ll.75-78).

Method: sampling procedure, study time are still vague here. Did you used any exclusion criteria? Please provide a justification/ rationale for the sample size. Please also provide the attrition rate. How did you handle with missing data? The method of estimation for the CFA is not clear. Which software did you use for conducting CFA?

🡪 Thank you for these comments. We described the sampling procedure in more detail mentioning the exclusion criteria and the justification for the sample size. We also included information on the handling of dropout rates and missing values. The CFA method was maximum likelihood estimation. Information regarding software used for each analysis is also included (p.5 ll. 88-96, p.7 ll. 143-150).

Results: please adjust your P-values for multiple comparisons

🡪 Authors appreciate this important advice. We adjusted our P-values for multiple comparisons, applying the Bonferroni correction (p.7 ll.143-144); as a result, the association between FCV-19S-J and living place in Factor 1 and Factor 2 is no longer significant (p.11 l. 203).

Finally, following the advice of the academic editors, we have made the corrections to the text and added necessary information as follows.

1. We ensured that our manuscript meets PLOS ONE's style requirements, including those for file naming.

2. We provide additional details regarding participant consent (p.8, l. 153-155).

3. The lead author’s ORCID iD has been added and validated in Editorial Manager.

4. We added captions for our Supporting Information files at the end of our manuscript (p.27 l.500).
